# Systematic Review on the Importance of Gut Microbiota in the Regulation of Type 2 Diabetes Through Physical Activity and Exercise

**DOI:** 10.3390/cimb47070505

**Published:** 2025-07-01

**Authors:** Luis Muguerza-Rodríguez, Alba Mier, Jesus G. Ponce-González, Cristina Casals, Juan Corral-Pérez

**Affiliations:** 1 ExPhy Research Group, Department of Physical Education, Instituto de Investigación e Innovación Biomédica de Cádiz (INiBICA), Universidad de Cádiz, 11519 Cádiz, Spain; luis.muguerzarodriguez@alum.uca.es (L.M.-R.); alba.mierperulero@alum.uca.es (A.M.); jesusgustavo.ponce@uca.es (J.G.P.-G.); juan.corral@uca.es (J.C.-P.); 2 School of Health Sciences, International University of La Rioja, 26001 Logroño, Spain

**Keywords:** obesity, intestinal dysbiosis, metabolic diseases, insulin resistance

## Abstract

Type 2 diabetes (T2D) is a major global health issue, influenced by sedentary behavior and obesity. Emerging evidence implicates the gut microbiota in T2D pathophysiology through effects on glucose metabolism, inflammation, and insulin sensitivity. This systematic review included eleven studies, six observational and five interventional, examining the relationship between physical activity, exercise, and gut microbiota in individuals with or at risk of T2D. Observational studies associated low physical activity and high sedentary time with reduced α-diversity and increased abundance of potentially harmful bacteria. Interventional studies showed that structured exercise, including moderate-intensity and sprint interval training, increased beneficial bacteria such as *Faecalibacterium*, *Veillonella*, *Lachnospira*, and *Bifidobacterium*, linked to anti-inflammatory effects and improved metabolic profiles. However, overall microbial diversity often remained unchanged unless combined with dietary modifications. Exercise also reduced levels of trimethylamine N-oxide, a metabolite linked to cardiovascular risk. Despite increases in butyrate-producing taxa, most studies did not report significant short-term changes in short-chain fatty acid levels, highlighting the complex interaction between microbiota and host metabolism. These findings support physical activity and exercise as modifiable factors that can influence gut microbiota composition, potentially contributing to improved metabolic regulation and better management of T2D.

## 1. Introduction

The human intestine harbors billions of microorganisms, including bacteria, protozoa, viruses, and archaea, collectively forming a complex ecosystem known as the gut microbiota. This microbial community plays a vital role in maintaining human health by modulating immune and inflammatory responses, regulating neuronal signaling, preserving intestinal barrier integrity, and contributing to the biosynthesis of vitamins, steroid hormones, and neurotransmitters. Moreover, the gut microbiota significantly influences the metabolism of branched-chain and aromatic amino acids, bile acids, and various drugs [1]. The composition of the human microbiome varies widely among individuals and is shaped by ethnicity, genetics, environment, diet, and early microbial exposure. Six dominant bacterial phyla are commonly observed: *Actinobacteria*, *Bacillota*, *Bacteroidetes*, *Fusobacteria*, *Verrucomicrobia*, and *Proteobacteria* [2].

The gut microbiota is mainly analyzed using microbial DNA from fecal samples via 16S rRNA gene amplicon or shotgun metagenomic sequencing, with the latter providing higher resolution data [3,4]. These methods measure α-diversity, reflecting species richness and abundance within samples, and β-diversity, showing differences between samples. Metagenomics can also assess metabolic outputs of the microbiota. Dysbiosis, or an imbalance in the microbiota, reduces short-chain fatty acid (SCFA) synthesis, important for gut health, β-cell proliferation, and insulin biosynthesis [5]. Dysbiosis also affects trimethylamine production, as it is converted to trimethylamine N-oxide (TMAO), a metabolite linked to increased type 2 diabetes (T2D) risk [1,6].

T2D affects over 500 million people worldwide and is characterized by impaired insulin secretion and resistance, causing hyperglycemia [7,8]. This figure is projected to increase, particularly in low-income countries [8]. T2D is a leading cause of death globally, with many cases in younger populations [7]. Although no consensus exists on gut microbiota composition in T2D, common findings include a higher *Bacillota*/*Bacteroidetes* ratio and increased *Blautia* abundance in patients compared to healthy controls [1,9]. Decreased butyrate-producing bacteria, such as *Faecalibacterium* and *Roseburia,* also appear central to T2D development [10]. Beneficial taxa like *Alistipes*, *Akkermansia*, and *Haemophilus*, linked to anti-inflammatory effects, gut barrier reinforcement, and glucose tolerance, are generally reduced in T2D [11].

Given the link between gut microbiota and T2D pathophysiology, lifestyle interventions like exercise are crucial to explore. Exercise improves glycemic control and insulin sensitivity; for example, a single session of high-intensity exercise can increase glucose uptake by at least 40%, improve glucose tolerance, and enhance insulin sensitivity within 12 to 48 h post-workout [12,13,14]. Aerobic training boosts short-term insulin sensitivity and enhances mitochondrial function, while resistance training increases muscle mass, bone density, and insulin sensitivity, especially when combined with caloric restriction [15,16,17]. Combined aerobic and resistance training may yield even greater benefits for glucose regulation [17].

Beyond metabolic effects, exercise and physical activity influence the gut microbiota by reducing gastrointestinal transit time and promoting beneficial bacteria, particularly butyrate producers like *Roseburia hominis* and *Faecalibacterium prausnitzii* [12,13,18]. This modulation strengthens the intestinal barrier, reduces inflammation, and improves the *Bacteroidota*/*Bacillota* ratio associated with weight control and gut health [14,19,20,21]. Moderate to vigorous intensity exercise appears most effective for these microbial benefits [20].

Despite promising findings, heterogeneity in study designs and exercise protocols limits conclusions. This systematic review therefore synthesizes current evidence on the effects of physical exercise on gut microbiota composition and its potential role in managing T2D. The review includes observational and interventional studies assessing microbiota changes in response to exercise interventions.

## 2. Materials and Methods

### 2.1. Protocol Registration and Literature Search Methodology

This systematic review was registered with the International Prospective Register of Systematic Reviews (PROSPERO; registration no. CRD420251052730) and conducted in accordance with the Preferred Reporting Items for Systematic Reviews and Meta-Analyses (PRISMA) guidelines.

A scientific literature review was conducted using two electronic databases, PubMed and Web of Science, without applying time restrictions.

The search equation used in PubMed was (Exercise OR “Physical activity” OR “Physical exercise” OR Training AND (Microbiota OR “Gastrointestinal Microbiomes” OR “Gut Microbiome” OR “Gut Microflora” OR “Gut microbiota” OR “Gastrointestinal Flora” OR “Gastrointestinal Microflora” OR “Intestinal microbiome” OR “Intestinal microbiota” OR “Intestinal microflora” OR “Enteric bacteria”) AND (“Diabetes mellitus type 2” OR “Ketosis-Resistant Diabetes Mellitus” OR “Diabetes Mellitus, Non Insulin Dependent” OR “Diabetes Mellitus, Non-Insulin-Dependent” OR “Non-Insulin-Dependent Diabetes Mellitus OR Diabetes Mellitus, Type II” OR “Diabetes Mellitus, Noninsulin Dependent” OR “Type 2 Diabetes Mellitus” OR “Noninsulin-Dependent Diabetes Mellitus” OR “Noninsulin Dependent Diabetes Mellitus” OR “Type 2 Diabetes OR Diabetes, Type 2”).

The search equation used in Web of Science was ((TS = (Exercise OR “Physical activity” OR “Physical exercise” OR “Exercise training”)) AND TS = (Microbiota OR “Gastrointestinal Microbiomes” OR “Gut Microbiome” OR “Gut Microflora” OR “Gut microbiota” OR “Gastrointestinal Flora” OR “Gastrointestinal Microflora” OR “Intestinal microbiome” OR “Intestinal microbiota” OR “Intestinal microflora” OR “Enteric bacteria”)) AND TS = (“Diabetes mellitus type 2” OR “Ketosis-Resistant Diabetes Mellitus” OR “Diabetes Mellitus, Non Insulin Dependent” OR “Diabetes Mellitus, Non-Insulin-Dependent” OR “Non-Insulin-Dependent Diabetes Mellitus” OR “Diabetes Mellitus, Type II” OR “Diabetes Mellitus, Noninsulin Dependent” OR “Type 2 Diabetes Mellitus” OR “Noninsulin-Dependent Diabetes Mellitus” OR “Noninsulin Dependent Diabetes Mellitus” OR “Type 2 Diabetes” OR “Diabetes, Type 2”)”.

### 2.2. Inclusion/Exclusion Criteria

#### 2.2.1. Inclusion Criteria

The inclusion criteria were defined according to the PICO (Population, Intervention, Comparison, Outcome) methodology:

- Population: We included studies involving humans with prediabetes, T2D, or individuals at risk of developing type 2 diabetes.
- Intervention: Eligible interventions comprised cross-sectional studies analyzing gut microbiota through fecal sample collection and assessing physical activity via accelerometry or questionnaires. Experimental studies implementing a structured exercise program were also included.
- Comparison: Comparison of gut microbiota among individuals with different levels of physical activity or between groups that underwent an exercise intervention and those that did not.
- Outcome: Physical activity level, blood biomarkers, fecal microbiome composition, insulin resistance, and cardiorespiratory fitness (VO_2max_).

#### 2.2.2. Exclusion Criteria

Regarding the exclusion criteria, studies that did not measure physical activity or exercise, as well as individuals who were taking prebiotic or probiotic supplementation, were excluded from the review.

### 2.3. Screening Process

The search results were imported into the Mendeley Reference Manager platform to review and remove duplicate documents. The initial screening of titles and abstracts was conducted independently and blindly by both researchers. In cases where no clear consensus was reached, the principal investigator made the final decision on inclusion or exclusion.

### 2.4. Data Extraction

The selected studies underwent a full-text review for data extraction. A dedicated data extraction form was developed to collect key trial information, including author, year of publication, population characteristics, age, clinical condition, study type, type of intervention, sample size, training modality and intensity, volume, duration of the intervention, and weekly training frequency. Outcome values were considered at the end of the intervention period, regardless of the total study duration. Data was independently extracted by two researchers, who also conducted a cross-validation. Subsequently, a third researcher reviewed all the collected information to ensure the consistency and accuracy of the values.

## 3. Results

A total of 580 studies were collected: 171 from PubMed and 409 from Web of Science. Applying the “Humans” filter in both databases automatically excluded 118 studies (42 from PubMed and 76 from Web of Science) that had been conducted on animals. Additionally, six studies were removed as duplicates, and one was excluded for being marked as ineligible by automation tools.

Of the remaining 455 studies, 208 were excluded for not meeting the required design criteria (cross-sectional or clinical trials studies), leaving 247 studies from both databases. None were excluded due to retrieval issues.

Among those 247 studies, 201 were excluded for not presenting results on fecal microbiota composition, and 35 were excluded for not including a diabetic population or individuals with characteristics associated with developing diabetes.

**Figure 1:**
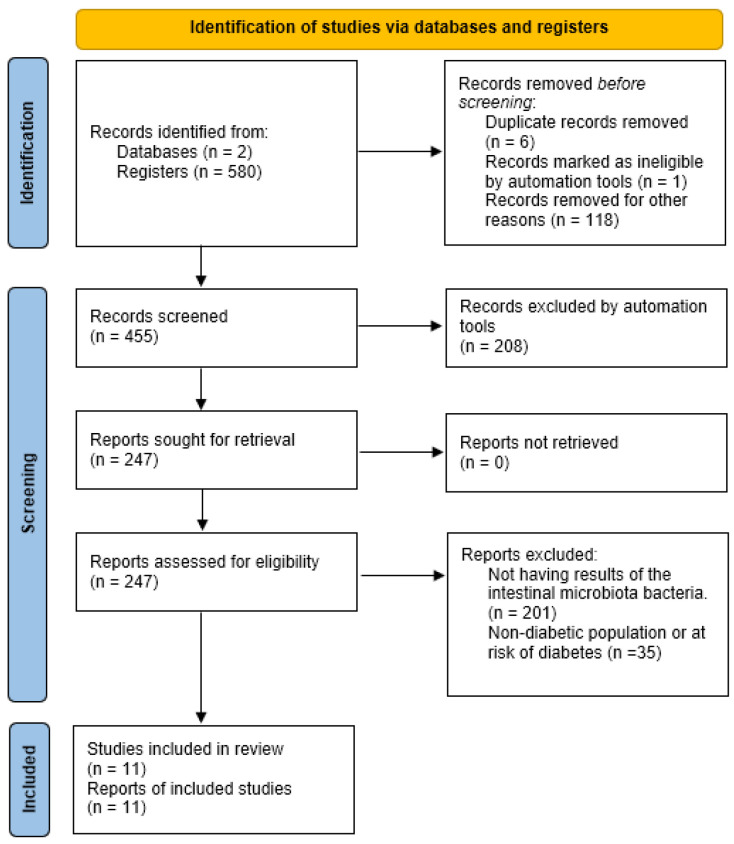
Preferred Reporting Items for Systematic Reviews and Meta-Analyses (PRISMA) 2020 flow diagram, including searches of databases, registers, and other resources.

Ultimately, 11 articles were included in the systematic review. A synthesis of this process can be seen in Figure 1.

Of the 11 articles included in this systematic review, 6 were cross-sectional studies and 5 were studies that included experimental protocols. Both study designs are represented in Table 1 and Table 2, respectively.

### 3.1. Observational Evidence on Physical Activity and Gut Microbiota in T2D

#### 3.1.1. Physical Activity

Two observational studies assessed the relationship between physical activity levels and gut microbiota composition in adults with or at risk of T2D. Houttu et al. [25] reported that physically active individuals exhibited a higher abundance of *Lachnospiraceae* and *Veillonella* (*Bacillota*), whereas sedentary individuals had elevated levels of *Roseburia hominis*, *Erysipelatoclostridium*, *Lachnoclostridium*, and members of the *Bacteroidetes* phylum, particularly *Prevotella_2*. Predictive microbial signatures of physical activity were distributed across the dominant phyla *Bacillota*, *Bacteroidetes*, *Proteobacteria*, and *Actinobacteria*, and other types of phyla such as *Lentisphaerae*.

Similarly, Antush et al. [22] found that higher sedentary behavior correlated with lower α-diversity and a higher *Bacillota*/*Bacteroidetes* ratio, both markers previously linked with metabolic dysfunction.

#### 3.1.2. Circulating TMAO and Physical Activity

Two studies explored the impact of physical activity on levels of TMAO, a gut-derived metabolite associated with T2D risk. Argyridou et al. [23] demonstrated that moderate to vigorous physical activity was associated with reduced TMAO levels. Kalagi et al. [26] confirmed that elevated TMAO was linked to T2D and identified lower levels of *Clostridiaceae*, *Peptostreptococcaceae*, *Clostridium*, *Itestinibacter*, and *Romboutsia* in individuals with T2D, suggesting potential microbial targets influenced by physical activity.

#### 3.1.3. Microbial Diversity and Insulin Resistance

Only one observational study [24] reported that greater α- and β-microbial diversity were inversely associated with insulin resistance. Specifically, the *Ruminococcaceae NK4A214* group was positively correlated with improved insulin sensitivity, reinforcing the metabolic relevance of microbial diversity in physically active populations.

#### 3.1.4. Body Composition and Gut Microbiota

Somnuk et al. [27] examined gut microbiota in relation to body weight and found that overweight individuals had a higher abundance of *Clostridiaceae-1*, *Bacillaceae-1*, and *Wohlfahrtiimonas*. In contrast, *Prevotella* was more prevalent in individuals with normal weight, while taxa such as *Eggerthellaceae*, *Rikenellaceae*, *Nocardioidaceae*, and *Chitinophagaceae* were depleted in those with obesity.

**Table 2:** Interventional studies: effects of exercise type and intensity on gut microbiota.

Reference	Population	Intervention	Measured Variables	Main Findings
Beals et al. (2023) [28]	- Diet group: N = 8 insulin-resistant women. - Age: 43.0 ± 3.0 years. - Diet + exercise group: N = 8 insulin-resistant women. - Age: 46.0 ± 3.0 years.	- Plant-based diet (70% CHO, 15% fat, 15% protein). - Exercise: 6 sessions/week (4 supervised, 2 at home) 1. HIIT (10 × 1 min all-out/1 min < 50% HRmax). 2. Strength training (alternating intensity/volume).	- Gut microbiota (16S rRNA sequencing). - Plasma proteome (SomaScan). - Skeletal muscle transcriptome (RNA-seq). - Insulin sensitivity (OGTT).	- No changes in α-diversity. - ↑ β-diversity in both groups.
Motiani et al. (2020) [29]	- N = 26 (N = 9 (1 woman) prediabetic, N = 17 (6 women) type 2 diabetic). - Age: 49.0 ± 4.0 years.	- SIT: 30 sec all-out intervals/4 min rest; 4–6 intervals - MICT: 40–60 min cycling at 60% VO_2max_	- Glucose and fatty acid uptake (PET). - Gut microbiota (16S rRNA sequencing). - Serum inflammatory markers (multiplex and ELISA).	- Both groups: ↓ *Bacillota*/*Bacteroidetes* ratio (↑ *Bacteroidetes*) ↑ *Blautia* and *Clostridium*. - SIT group: ↑ *Lachnospira*. - MICT group: ↑ *Veillonella*, *Veillonella dispar*, and *Faecalibacterium*. - No significant changes in richness/diversity between groups.
Torquiati et al. (2023) [30]	- N = 14 T2D sedentary individuals divided group (N = 7) or MICT group (N = 7). - Age: 64.3 ± 6.4 years.	- HIIT: 8 × 1 min (85–95% peak HR). - MICT: 52.5 min/session, 4×/week. - 2 sessions low/moderate intensity, 2 moderate only.	- Gut microbiota (abundance, functional profile, SCFAs). - HbA1c, fasting glucose, lipids (Randox Daytona+). - Diet (FoodWorks).	- HIIT group: ↑ butyrate producers from the *Rypelothrichales*, *Oscillospirales orders*, *M. smithii* and *Negativibacilli* spp. - MICT group: ↑ *Bifidobacterium* and *Escherichia A. municiphila*, and butyrate-producing taxa from the *Lachnospirales* order and *Clostridium cluster IV*. - No significant changes in SCFAs.
Verheggen et al. (2021) [31]	- N = 14 (7 women) in risk of T2D. - Age: 51.0 ± 11.0 years.	- Exercise session: 5 min warm-up + 50 min at 65–85% HRR + 5 min cool-down.	- Insulin sensitivity (euglycemic clamp). - Cardiovascular fitness (VO_2max_). - Visceral fat (DEXA). - Gut microbiota (16S rRNA sequencing).	- ↑ Insulin sensitivity and ↓ visceral fat. - No changes in α-/β-diversity. - ↑ *Ruminococcus gauvreauii*, *Lachnospiraceae FCS020* group, and *Anaerostipes*. - *R. gauvreauii* positively correlated with VO_2max_.
Wei et al. (2022) [32]	- N = 98 (46 women) with T2D. - Age: 54.3 ± 8.9 years.	- Intervention: 5–6 aerobic sessions/week, 2–3 with strength + hypocaloric diet (45–65% CHO, 15–20% protein, 20–35% fat). - Control: diabetes education + blinded medical care.	- Diet (FFQ, intervention group only). - Gut microbiota (16S rRNA sequencing).	- Changes in α-diversity not correlated with clinical variables. - Gut microbiota did not reflect treatment-induced clinical changes.

Abbreviations: DEXA: dual-energy X-ray absorptiometry; ELISA: enzyme-linked immunoassay kit; FFQ: Food Frequency Questionnaire; HbA1c: glycated hemoglobin; HIIT: high-intensity interval training; HR: heart rate; MICT: moderate-intensity continuous training; N: number of persons; PET: positron emission tomography; SCFAs: short-chain fatty acids; SIT: sprint interval training; VO_2max_: maximum oxygen uptake.

### 3.2. Interventional Studies: Effects of Exercise Type and Intensity on Gut Microbiota

#### 3.2.1. Type and Intensity of Exercise

Two trials compared different exercise modalities. Motiani et al. [29] found that both Sprint Interval Training (SIT) and Moderate-Intensity Continuous Training (MICT) reduced the *Bacillota/Bacteroidetes* ratio—largely due to an increase in *Bacteroidetes*—and decreased levels of *Blautia* and *Clostridium*. SIT promoted the growth of *Lachnospira*, whereas MICT led to increases in *Veillonella*, *Veillonella dispar*, and *Faecalibacterium*. Both protocols also increased levels of beneficial genera like *Bifidobacterium* and *Escherichia A. municiphila*.

Torquati et al. [30] reported that MICT increased butyrate-producing taxa such as *Lachnospirales* and *Clostridium cluster IV*, while High-Intensity Interval Training (HIIT) enhanced the presence of *Rysipelotrichales* and *Oscillospirales*—microbial groups potentially linked to improved metabolic health.

#### 3.2.2. Short-Chain Fatty Acids (SCFAs)

Torquati et al. [30] was the only study to assess SCFAs, reporting no significant post-intervention changes in SCFA concentrations. Nevertheless, both HIIT and MICT increased the abundance of butyrate-producing taxa, suggesting a potential for longer-term metabolic benefits.

#### 3.2.3. Microbial Diversity

Four intervention studies [28,29,31,32] investigated microbial diversity following exercise and generally reported no significant changes in α- or β-diversity. However, Beals et al. [28] noted a shift in β-diversity, although the effect was not attributed solely to exercise, suggesting that dietary confounding may influence diversity outcomes.

#### 3.2.4. Gut Microbiota Composition

Three studies evaluated taxonomic shifts after exercise interventions. Verheggen et al. [31] observed increases in *Ruminococcus gauvreauii*, *Lachnospiraceae FCS020* group, and *Anaerostipes*, with positive correlations between *R. gauvreauii* and VO_2max_, indicating a link between aerobic fitness and microbial composition.

Motiani et al. [29] corroborated previous findings with SIT enhancing *Lachnospira* and MICT favoring *Veillonella* and *Faecalibacterium*. Both exercise types also contributed to a favorable reduction in the *Bacillota/Bacteroidetes* ratio.

## 4. Discussion

This systematic review identified eleven studies, six observational and five interventional, examining the relationship between physical activity and gut microbiota composition in individuals with T2D or at risk of developing the disease. Despite heterogeneity in study populations, methodologies, and outcome measures, several consistent patterns emerged.

### 4.1. Observational Evidence on Physical Activity and Gut Microbiota in T2D

Cross-sectional studies consistently reported that lower levels of physical activity and higher sedentary behavior were associated with unfavorable gut microbiota profiles in adults with or at risk of T2D. For example, sedentary individuals showed an increased abundance of potentially pathogenic taxa such as *Escherichia*/*Shigella* and reduced levels of beneficial bacteria like *Lachnospiraceae* and *Veillonella*, microbial groups linked to anti-inflammatory functions and metabolic health. These findings are in line with evidence that physical inactivity can promote gut dysbiosis, which may exacerbate insulin resistance and systemic inflammation [22,25]. From a physiological perspective, this microbial imbalance may impair glucose uptake, increase gut permeability, and elevate inflammatory cytokines, worsening glycemic control and promoting T2D progression. Recent research has shown that gut-derived inflammation can also contribute to impaired mitochondrial function in skeletal muscle, further diminishing glucose disposal [33].

α-diversity, often considered a proxy for microbial resilience, was consistently lower among less active individuals. Notably, Antush et al. [22] reported a direct correlation between sedentary time and a higher *Bacillota*/*Bacteroidetes* ratio, a microbial marker commonly associated with metabolic disorders. Similarly, Chen et al. [24] linked higher microbial diversity with lower insulin resistance, suggesting that even in observational settings, microbial richness may mediate the beneficial effects of physical activity on glucose metabolism. Clinically, this supports the idea that promoting physical activity may not only improve cardiometabolic profiles but also enhance gut microbial balance, potentially delaying the onset or progression of T2D. These associations align with the concept of the gut–muscle axis, where microbial composition may impact insulin signaling and muscle glucose uptake [34].

The metabolite TMAO, a gut-derived metabolite implicated in cardiovascular and metabolic diseases, emerged as a relevant outcome in two studies. Argyridou et al. [23] found that moderate-to-vigorous physical activity was associated with reduced TMAO levels, while Kalagi et al. [26] showed that the association between TMAO and T2D risk lost significance after adjusting for physical activity and diet. These results suggest that TMAO levels may not only reflect dietary intake but also lifestyle-related microbiota changes, offering a potential biomarker for tracking microbiota–host interactions in response to physical activity. Physiologically, lower TMAO levels may reduce endothelial dysfunction, oxidative stress, and chronic inflammation—all of which are implicated in the pathogenesis of T2D and its vascular complications [35]. Clinically, tracking TMAO could help identify individuals at higher cardiometabolic risk and monitor the efficacy of lifestyle interventions, including exercise. In addition, elevated TMAO concentrations have been associated with impaired insulin signaling and pancreatic β-cell dysfunction, which are central features of T2D pathophysiology [36].

### 4.2. Interventional Studies: Effects of Exercise Type and Intensity on Gut Microbiota

Interventional studies provide more robust causal evidence that exercise influences gut microbial composition. Both aerobic and resistance-based exercise protocols induced taxonomic changes, although they did not consistently alter microbial diversity. Exercise decreases the *Bacillota*/*Bacteroides* ratio, providing multiple benefits for individuals with T2D. Motiani et al. [29] showed that both HIIT and SIT reduced the *Bacillota*/*Bacteroides* ratio in individuals with prediabetes and T2D. However, Wei et al. [32] reported no significant differences in gut microbiota between a lifestyle intervention and standard care interventions. This discrepancy may be explained by the fact that the standard care group was not monitored for diet or daily physical activity, potentially introducing a confounding variable. Nonetheless, it is worth noting that beneficial changes were observed between the start of the intervention and after 12 months.

Additionally, Motiani et al. [29] and Torquati et al. [30] demonstrated that both SIT and MICT increased the relative abundance of beneficial taxa such as *Veillonella*, *Faecalibacterium*, and butyrate producers like *Lachnospira* and *Anaerostipes*. These microbes are known to produce SCFAs, which enhance gut barrier function and improve insulin sensitivity [29,30]. From a physiological standpoint, SCFAs contribute to energy metabolism, promote GLP-1 secretion, regulate immune responses, and inhibit histone deacetylases, all critical in preventing or managing insulin resistance [37]. The increased abundance of SCFA producers may therefore underline some of the glycemic improvements observed in exercise trials. Clinically, identifying individuals with a low baseline abundance of these taxa may help personalize exercise prescriptions to enhance microbiota responsiveness and optimize metabolic outcomes. Further, some taxa enriched through exercise, such as *Faecalibacterium prausnitzii*, have been inversely associated with HbA1c levels and pro-inflammatory markers in patients with T2D [38].

Only one study [30] assessed short-chain fatty acids directly, reporting no significant changes in SCFA concentrations post-intervention. However, the increase in butyrate-producing taxa in both HIIT and MICT groups suggests the potential for longer-term benefits not captured within the study duration [30]. Since SCFAs play critical roles in glucose homeostasis, lipid metabolism, and inflammation regulation, future studies should combine taxonomic profiling with metagenomics or metabolomics to evaluate functional microbial outputs in response to exercise. Physiologically, SCFAs act as signaling molecules that influence insulin sensitivity, adipose tissue metabolism, hepatic glucose production, and gut–brain axis signaling. Clinically, enhancing SCFA production through exercise may reduce the need for pharmacologic glycemic control and lower systemic inflammation, important goals in T2D management. Importantly, butyrate has been shown to enhance mitochondrial function and improve glucose uptake in skeletal muscle [39].

Interestingly, the magnitude and direction of microbial shifts varied by exercise type. SIT appeared to favor the growth of *Lachnospira* and *Clostridium* species, whereas MICT enriched *Veillonella* and *Bifidobacterium*, suggesting that microbial responses to exercise may be intensity- and modality-dependent. This distinction could have practical implications for tailoring exercise prescriptions to optimize gut health in people with or at risk of T2D.

Contrary to expectations, most intervention studies failed to show significant changes in α- or β-diversity following exercise, even in protocols lasting several weeks. Beals et al. [28] reported some shifts in β-diversity, but effects could not be disentangled from dietary influences. These inconsistencies may reflect the inherent stability of microbial diversity in adults, requiring longer interventions or more intense stimuli to yield measurable change. Alternatively, taxonomic shifts may be more functionally relevant than diversity indices alone, highlighting the need for integrative analyses that include microbial metabolites and host metabolic endpoints. From a physiological and clinical standpoint, the lack of diversity change does not necessarily imply a lack of benefit. It suggests instead that compositional and functional changes may precede or substitute for shifts in diversity, especially in the short term. Clinically, functional redundancy in microbial ecosystems may allow for resilience in metabolic pathways even when diversity metrics remain stable.

### 4.3. Limitations of This Systematic Review

This review has several limitations. First, the number of eligible studies was small, reflecting the novelty of the field. Second, considerable methodological heterogeneity existed in microbiota sequencing methods, physical activity assessments, and population characteristics, limiting cross-study comparability. Third, only a few studies controlled dietary intake, a critical confounder in microbiota research.

Nevertheless, these findings suggest promising directions for future research. Standardized protocols incorporating diet-controlled interventions, metagenomic sequencing, and longitudinal designs are needed to clarify the causal links between exercise, gut microbiota, and metabolic health. Additionally, stratifying analyses by sex, medication use, and metabolic status may reveal subpopulations most responsive to microbiota-modulating effects of physical activity. From a clinical standpoint, this line of research may help integrate microbiome profiling into personalized lifestyle interventions for T2D prevention and treatment. Trials could also explore synergistic interventions combining diet, prebiotics, or fecal microbiota transplantation with exercise to enhance metabolic outcomes.

Over the years, approximately 2000 articles have been published exploring the relationship between gut microbiota and type 2 diabetes. However, when narrowing the focus to studies in which physical exercise is the primary variable, only five clinical trials were identified and included in this review. This highlights a clear gap in the literature and underscores the need for further research to elucidate the role of physical exercise, considering its key components such as volume, type, intensity, and frequency, in modulating gut microbiota and its impact on the regulation of T2D.

## 5. Conclusions

In summary, this systematic review indicates that physical activity, particularly when structured and sustained, can beneficially modulate gut microbiota composition in individuals with or at risk of T2D. While changes in microbial diversity were inconsistent, several taxa associated with improved metabolic outcomes were enriched through exercise, suggesting a role for the gut microbiome as a mediator of exercise benefits. These insights support the integration of exercise as a non-pharmacological strategy to optimize gut health and metabolic control in the context of diabetes prevention and management. Clinically, these findings underscore the potential of exercise as an adjunctive therapy to current pharmacologic regimens for T2D, particularly when combined with strategies that target gut microbial function and composition.

## Figures and Tables

**Table 1:** Observational evidence on physical activity and gut microbiota in T2D.

Reference	Population	Measured Variables	Main Findings
Antush et al. (2024) [22]	- N = 47 (29 women). - Healthy, pre-diabetic, and T2D. - Age: 51.0 ± 16.1 years.	- Physical activity (IPAQ). - Gut microbiota (16S rRNA sequencing).	- Sedentary behavior associated with ↓ α-diversity. - Positive correlation between sedentary behavior and *Bacillota*/*Bacteroidetes* ratio.
Argyridou et al. (2020) [23]	- N = 483 (167 women). - High risk of T2D. - Age: 63.5 ± 7.3 years.	- Physical activity (ActiGraph accelerometer). - TMAO (fasting blood sample).	- Moderate-to-vigorous physical activity associated with ↓ TMAO levels.
Chen et al. (2021) [24]	- N = 2166 (920 women). - T2D. - Age: 53.5 ± 8.6 years.	- Insulin resistance (glucose/insulin assays). - Gut microbiota (16S rRNA sequencing).	- ↑ α-diversity (Shannon richness) associated with ↓ insulin resistance. - β-diversity was associated with insulin resistance. - 7 taxa (*Christensenellaceae*, *Christensenellaceae group R7*, *Marvinbryantia*, *Ruminococcaceae UCG005*, *Ruminococcaceae UCG008*, *Ruminococcaceae UCG010*, and the *Ruminococcaceae NK4A214*) linked to ↓ insulin resistance and ↓ T2D risk. - ↑ *Clostridiaceae*, *Peptostreptococcaceae*, *Clostridium*, *Itestinibacter*, and *Romboutsia* linked to ↓ T2D risk.
Houttu et al. (2021) [25]	- N = 1334 (687 women). - General population stratified into sedentary (441/100) and active (868/62) by self-report and accelerometer physical activity, respectively. - Age: 51.0 ± 10.8 years.	- Physical activity (self-report and ActiHeart accelerometer). - Gut microbiota (16S rRNA MiSeq, V4 region).	- Sedentary group: ↑ *Proteobacteria* (*Enterobacteriales* *Enterobacteriaceae*, *Escherichia*/*Shigella*, and *Klebsiella*), ↑ *Bacteroidetes* (*Prevotella_2*), and *Bacillota* (*Roseburia hominis*, *Erysipelatoclostridium*, and *Lachnoclostridium*). - Active group: ↑ *Bacillota* (e.g., *Lachnospiraceae*, *Veillonella*). - ↑ *Bacillota*/*Bacteroidetes*, *Proteobacteria*, *Actinobacteria*, and *Lentisphaerae* (*Blautia* and *Lachnospiraceae*) predicted ↑ physical activity. - ↓ Arginine metabolism in active group, correlated with *Enterobacteriales*, *Enterobacteriaceae*, *Escherichia*/*Shigella*, *Klebsiella*, *Proteobacteria*, and *Veillonella*.
Kalagi et al. (2022) [26]	- N = 297 (259 women). - Healthy controls and T2D. - Age: 37 (33–45) and 55 (50–62) years.	- Physical activity (IPAQ). - Blood biomarkers (fasting blood samples).	- Unadjusted: ↑ TMAO associated with ↑ T2D risk. - After adjusting for physical activity and diet, the association was not significant.
Somnuk et al. (2023) [27]	- N = 60 (30 women). - Normal weight and overweight. - Age: 30.0 ± 9.0 years.	- Biochemical markers (fasting blood samples). - Lifestyle (IPAQ). - Gut microbiota (16S rRNA sequencing).	- Overweight group: ↑ *Clostridiaceae-1*, *Bacillaceae-1*, and *Wohlfahrtiimonas* (family level), *Clostridium sensu stricto*, *Senegalimassilia*, *Enterobacter*, *Citrobacter*, *Bacillus*, *Paraclostridium*, and *Lancefieldella*. - Overweight group: ↓ *Eggerthellaceae*, *Rikenellaceae*, *Nocardioidaceae*, *Chitinophagaceae*, *Alistipes*, *Fecalicatena*, *Oscillibacter*, *Limosilactobacillus*, *Slackia*, *Ruthenibacterium*, *Gordonibacter*, and *Longibaculum*. - Overweight group: ↑ sedentary time, ↓ physical activity.

Abbreviations: IPAQ: International Physical Activity Questionnaire; N: Number of participants; T2D: Type 2 diabetes; TMAO: Trimethylamine-N-oxide.

## Abbreviations

The following abbreviations are used in this manuscript:

BMI	Body Mass Index
DNA	Deoxyribonucleic Acid
HbA1c	Hemoglobin A1c
HIIT	High-Intensity Interval Training
MICT	Moderate-Intensity Continuous Training
N	Total participants
PRISMA	Preferred Reporting Items for Systematic Reviews and Meta-Analyses
rRNA	Ribosomal Ribonucleic Acid
SCFAs	Short-Chain Fatty Acids
SIT	Sprint Interval Training
TMAO	Trimethylamine N-oxide
TS	Topic Search
VO_2max_	Maximal oxygen uptake
